# The Dulness of Domestic Service

**Published:** 1891-05-23

**Authors:** 


### Editor's Letter-Box.

[Our correspondents are reminded that prolixity of statement is a great bar to publication, and that brevity of style and conciseness of statement greatly facilitate early insertion.]

### THE DULNESS OF DOMESTIC SERVICE.

Sir,—The writer of the Annotation which appeared in your paper of the 9th inst., on "The Dulness of Domestic Service," is, I think, mistaken about the want of interest taken by mistresses now in the welfare of their servants, for in order to keep one in the present day, we must give little work, good wages, and many "intervals of leisure." Whether the mistress be a conscientious person or not, this has become a matter of necessity. It seems more likely that the deterioration of domestic servants is due to their want of conscience and thoroughness, for whereas our mothers will tell us of loyalty, and even devotedness, in the past, we have now to be satisfied with "eye-service." I think this will remain so until "sweet reasonableness" asserts itself among domestic servants, and they, as well as we, try to understand the "give and take" of life.—Yours truly,

May 18, 1891. A Reasonable Mistress.

